# Concentration of Potentially Bioactive Compounds in Italian Extra Virgin Olive Oils from Various Sources by Using LC-MS and Multivariate Data Analysis

**DOI:** 10.3390/foods9081120

**Published:** 2020-08-13

**Authors:** Anna Różańska, Marina Russo, Francesco Cacciola, Fabio Salafia, Żaneta Polkowska, Paola Dugo, Luigi Mondello

**Affiliations:** 1 Department of Chemical, Biological, Pharmaceutical and Environmental Sciences, University of Messina, 98122 Messina, Italy; anna.rozanska@pg.edu.pl (A.R.); fsalafia@unime.it (F.S.); pdugo@unime.it (P.D.); lmondello@unime.it (L.M.); 2 Department of Analytical Chemistry, Faculty of Chemistry, Gdańsk University of Technology, 80-233 Gdańsk, Poland; zanpolko@pg.edu.pl; 3 Department of Biomedical, Dental, Morphological and Functional Imaging Sciences, University of Messina, 98122 Messina, Italy; cacciolaf@unime.it; 4 Chromaleont s.r.l., c/o Department of Chemical, Biological, Pharmaceutical and Environmental Sciences, University of Messina, 98122 Messina, Italy; 5 Department of Sciences and Technologies for Human and Environment, University Campus Bio-Medico of Rome, 00128 Rome, Italy; 6 BeSep s.r.l., c/o Department of Chemical, Biological, Pharmaceutical and Environmental Sciences, University of Messina, 98122 Messina, Italy

**Keywords:** extra-virgin olive oils, oleuropein, phenols, tocopherols, HPLC-MS, multivariate statistical data

## Abstract

High quality extra virgin olive oils represent an optimal source of nutraceuticals. The European Union (EU) is the world’s leading olive oil producer, with the Mediterranean region as the main contributor. This makes the EU the greatest exporter and consumer of olive oil in the world. However, small olive oil producers also contribute to olive oil production. Beneficial effects on human health of extra virgin olive oil are well known, and these can be correlated to the presence of vitamin E and phenols. Together with the origin of the olives, extraction technology can influence the chemical composition of extra virgin olive oil. The aim of this study was to investigate the concentration of potentially bioactive compounds in Italian extra virgin olive oils from various sources. For this purpose, vitamin E and phenolic fractions were characterized using high-performance liquid chromatography (HPLC) coupled with fluorescence, photodiode array and mass spectrometry detection in fifty samples of oil pressed at industrial plants and sixty-six samples of oil produced in low-scale mills. Multivariate statistical data analysis was used to determine the applicability of selected phenolic compounds as potential quality indicators of extra virgin olive oils.

## 1. Introduction

As reported by the European Commission [1] more than 69% of the world’s olive oil comes from the Mediterranean region, primarily from Spain. This makes the European Union (EU) the leading producer, exporter and consumer of olive oil in the world. The EU’s major producer member states are: Spain, Italy, Greece, France, Portugal, Croatia, Slovenia, Malta and Cyprus. However, between these nine nations Spain is the biggest producer, with 63% of the EU’s total production. In addition, a substantial contribution to the total EU olive oil production comes from Italy, Greece and Portugal, with a production of 17%, 14% and 5%, respectively. Extra virgin olive oil (EVOO) produced in the EU is subjected to marketing standards. These requirements ensure that consumers receive a product with a standardized and satisfactory quality, and on the other hand ensure equal economic conditions to EU producers [2].

Parallel to the EU industrial EVOO production there is a market of smaller olive oil producers. This kind of manufacturing respects all the requirements demanded by the EU. However, the smaller olive oil producers try to distinguish their extra virgin olive oils from the anonymity of industrial oil production.

Beneficial effects on human health of EVOO are well known, and these can be correlated with the presence of a well-balanced acidic composition, and especially to the presence of fat-soluble vitamin and hydrophilic phenolic compounds (vitamin E and phenols) [3,4,5,6]. Vitamin E is composed of eight isomers, and in EVOO is principally represented by *α*-tocopherol [7], that is the isomer with the greatest biological activity [8,9]. Hydrophilic phenolic compounds present in EVOO belong to different classes: secoiridoids, phenolic acids, phenolic alcohols, lignans and flavonoids [10]. Thanks to this qualitative bioactive molecule composition, EVOO can be considered as a functional food. Moreover, together with the health promoting qualities these molecules prolong the shelf life of the oil increasing its stability, and stabilize the oil during frying [11,12].

In literature, several research articles can be found concerning the characterization of bioactive molecules in EVOOs [13,14,15], and some of these reported their determination despite being applied to limited data samples [7,16,17,18,19,20,21,22,23]. Recently our research group published a research article on the content of bioactive antioxidant molecules among 186 high quality Italian extra virgin olive oils belonging to eleven regions [24]. Data obtained showed that there were some differences between different regions even though they were not so marked and significant; this highlighted how the cold extraction technology employed kept antioxidant content unaltered.

In other cases, olive extraction technology was found as a parameter which could influence the chemical composition of EVOOs [25]. Anyway, all the stages of the oil production can effect the bioactive molecule composition. For instance, parameters that influence the quality and quantity of phenols and vitamin E are olive collection, type of crusher, crushing speed and conditions, type of decanter and its regulation, and so on [26]. So, differences in antioxidant molecule composition not only depend on cultivar, quality, territory, climate, storage conditions, olive maturity index, but also on production techniques [16,26].

The aim of this study was to investigate the nutraceutical properties of Italian extra virgin olive oils from various sources, namely, small farms and local distribution centers. For this purpose, vitamin E and phenolic fractions were characterized using HPLC techniques coupled with fluorescence (FLD), photodiode array (PDA) and mass spectrometry (MS) detection in fifty samples of oil pressed at industrial plants and sixty-six samples of oil produced in low-scale mills. Multivariate statistical data analysis was used to determine the applicability of selected phenolic compounds as potential quality indicators of extra virgin olive oils.

To the best of our knowledge, this is the first research article that reports the bioactive molecule content and a principal components analysis (PCA) statistical correlation between vitamin E and phenols in a large array of Italian high quality EVOOs (66) produced in different regions and fifty commercially available EVOOs.

## 2. Materials and Methods

### 2.1. Materials and Reagents

Standards: α-, β-, γ-tocopherol and α-tocotrienol were purchased from Extrasynthese (Extrasynthese S.A., Genay Cedex, France); gallic acid, tyrosol, hydroxytyrosol, apigenin, luteolin, oleuropein and verbascoside were purchased from Merck Life Science (Merck KGaA, Darmstadt, Germany) as was the internal standard (ethyl gallate, IS). Solvents: acetonitrile, ethanol, formic acid, isopropanol, methanol and *n*-hexane used for chromatographic analysis were purchased from Merck Life Science (Merck KGaA, Darmstadt, Germany). Water (resistivity above 18 MΩcm) was obtained from a Milli-Q SP Reagent Water System (Merck KGaA, Darmstadt, Germany).

### 2.2. Samples

Sixty six Italian extra-virgin olive oils (marked as EVOOs) were collected from various Italian low-scale oil mills sited in: Abruzzo, Apulia, Calabria, Campania, Lazio, Liguria, Garda area (Lombardy, Trentino, Veneto), Sardinia, Sicily, Tuscany and Umbria. These samples were collected on the basis of the European extra quality olive oil award called “il Magnifico”. This award is assigned to the best producers of extra quality olive oil in Europe. Moreover, fifty commercially available extra-virgin olive oils (marked as COOs) were purchased from local shops and supermarkets labelled as samples from Italy, EU countries, EU/Italy and Sicily. As reported in commercial olive oil labels, both olive oil samples from EU countries and EU/Italy were processed by olive mills located in Italy, but olives were harvested from EU member states or Italy and other EU member states respectively. All information about the samples are listed in Table 1. The oils were poured into amber glass vials and stored at −20 °C, each sample was thawed and analyzed on the same day.

### 2.3. Tocopherols and Tocotrienols Determination by NP-HPLC-FLD

Tocopherols and tocotrienols were determined according to the method previously developed and validated by Dugo et al. [24]. Briefly, olive oils samples were diluted in n-hexane (1:10, 1:15 or 1:30 *v*/*v*). The HPLC analyses were carried out using a Shimadzu Nexera-X2 system (Shimadzu, Milan, Italy) equipped with an on-line degasser (DGU-20ASR), an autosampler (SIL-30 AC), two dual-plunger parallel-flow pumps (LC-30 AD), a column oven (CTO-20AC), a communication bus module (CBM-20A) and a fluorescence detector (RF-20AXS). Phenolic components were separated on a normal-phase Ascentis Si column (250 × 4.6 mm I.D. with 5 μm particle size, Merck KGaA, Darmstadt, Germany) under the following conditions: mobile phase n-hexane:isopropanol (99:1; *v*/*v*); flow rate (1.7 mg L^−1^); column oven temperature (25 °C) and injection volume (5 μL). The identification and quantification were conducted by using a fluorescence detector at an excitation wavelength of 290 nm and emission wavelength of 330 nm. Data acquisition was performed by the LCMS solution v. 5.85 software (Shimadzu, Milan, Italy).

To quantify the vitamin E content in the EVOOs sample calibration curves have been constructed by using each single available standard, according to the method previously developed and validated by Dugo et al. [24]. Briefly, five different concentrations of each component, in the range between 5 and 0.005 mg L^−1^, prepared by diluting a stock solution of about 100 mg L^−1^, were analyzed five consecutive times by NP-HPLC.

### 2.4. Phenols Determination by RP-HPLC-PDA/MS

Phenolic acid, phenolic alcohols, flavonoids and secoiridoids were determined using the method described by Dugo et al. [24]. Briefly, 1 mL of olive oil was diluted in 1 mL of n-hexane and homogenized. Next, the sample was extracted with 1 mL of H_2_O:methanol (2:3, *v*/*v*) solution for 2 min in an Elmasonic P 60H ultrasonic bath (Elma Schmidbaure GmbH, Singen, Germany), and centrifuged for 10 min at 3000 rpm. The polar phase was transferred and washed with 1 mL of n-hexane. To the final extract, 20 μL of ethyl gallate (1 mg mL^−1^ in methanol) was added. Sample extraction methods validation was carried out according to Dugo et al. [24] by using a sample of soybean oil added of known amounts of luteolin, oleuropein, apigenin, tyrosol and hydroxytyrosol.

The HPLC analyses were carried out using a Shimadzu Prominence LC-20A (Shimadzu, Kyoto, Japan) equipped with a degasser (DGU-20A5), an autosampler (SIL-20 A), two dual-plunger parallel-flow pumps (LC-20 AD), a communication bus module (CBM-20A), a photodiode array detector (SPD-M20A) and a single-quadrupole mass spectrometer (LCMS-2020) equipped with an electrospray ionization (ESI) source, operating in negative ionization mode. The separation was conducted on a reverse-phase Ascentis Express C18 column (150 × 4.6 mm, 2.7 μm, Merck Life Science, Merck KGaA, Darmstadt, Germany) under the following conditions: mobile phases A—0.1% acetic acid and B—acetonitrile with 0.1% formic acid; flow rate (1 mL min^−1^); column oven temperature (25 °C) and injection volume (5 μL). The gradients used were as follows: 0 min, 10% B; 4 min, 35% B; 12 min, 47% B; 12.5 min, 60% B; 16 min, 75% B; and 21 min, 100% B. Identification was carried out by both PDA (280 nm) and MS detection under the following conditions: mass spectral range (*m/z* 100–800); event time (0.5 s); nebulizing gas flow, N_2_ (1.5 L min^−1^); drying gas flow, N_2_ (5 L min^−1^); heat block temperature (300 °C); and desolvation line (DL) temperature (280 °C). Single ion monitoring (SIM) was used for quantification: gallic acid (170 *m/z*), tyrosol (138 *m/z*), hydroxytyrosol (154 *m/z*), apigenin (270 *m/z*), luteolin (286 *m/z*), oleocanthal (304 *m/z*), oleacein (320 *m/z*), oleuropein aglycone (378 *m/z*) and ligstroside aglycone (362 *m/z*). Data acquisition was performed by the LCMS solution v. 5.85 software (Shimadzu, Kyoto, Japan).

To quantify the hydrophilic phenols content in the one hundred and eighty-six high quality extra-virgin olive oil samples, the method previously described by Dugo et al. [24] was used. Briefly, five different concentrations of each component, in the range between 100 and 0.1 mg L^−1^, prepared by diluting a stock solution of about 1000 mg L^−1^ were analyzed five consecutive times by RP-HPLC. Before injection, 20 μL of internal standard (IS) ethyl gallate (1 mg mL^−1^) was added to 1 mL of each standard solution.

### 2.5. Multivariate Statistical Analysis

Each sample was analyzed in triplicate. One-way ANOVA using Tukey’s test was applied to evaluate the significant differences at a level of *p* < 0.05 among means (Statistica 12, StatSoft, Inc., Tulsa, OK, USA). The determined phenol concentration values in olive oil samples were used as input data for multivariate statistical data analysis using the dedicated Python toolset Orange v. 3.13 (Bioinformatics Lab, University of Ljubljana, Ljubljana, Slovenia). Initial data processing included standardization (centering by a mean value and scaling by standard deviation). Missing data (determined concentration below LOQ) was replaced by respective LOD/3 values. Analysis of variance within variables and feature selection was performed using ANOVA. During the research, various chemometric approaches were used, namely hierarchical cluster analysis (HCA) and principal components analysis (PCA) to differentiate samples of olive oil from various sources and k-nearest neighbors (k-NN) algorithm to classify oils according to their quality. HCA with Ward’s linkage was performed based on the Mahalanobis distance. To identify relevant clusters, 66% of the maximum distance between objects was used. Moreover, the concentration of seven phenols, which had the greatest impact on the result of a statistical analysis based on ANOVA, was used as input data to PCA. The PCA method made it possible to visualize the distinction between COO and EVOO samples. Classification accuracy and precision of supervised classification (k-NN) were validated using 10-fold cross-validation.

## 3. Results and Discussion

The results of the bioactive substance determination in extra virgin olive oils samples obtained from local Italian mills (EVOO) and from local distribution centers (COO) are summarized in Table 2. The results of EVOOs were divided into 11 Italian regions from which oils originated, while for COOs, 4 regions of origin were distinguished according to the information on the labels (see Table 1). Quantitative data obtained for Sicilian samples (EVOO and COO) were reported together in Table 2. In all the types of olive oil, 13 phenols (4 fat-soluble and 9 hydrophilic) were detected. The obtained results did not show any qualitative changes in the phenolic compound profile for olives of different geographical origin, as well as the process of their preparation.

**Figure 1:**
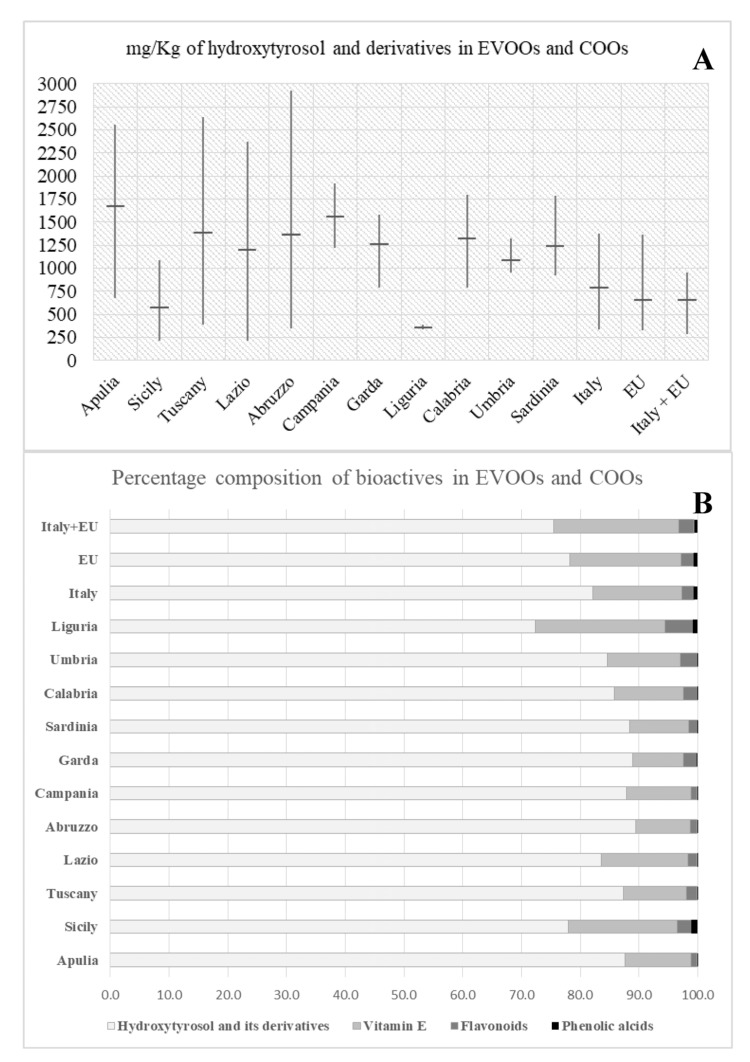
(**A**) mg kg^−1^ of hydroxytyrosol and its derivatives in EVOOs and COOs; (**B**) relative percentage of four chemical groups in EVOO and COO samples. For sample descriptions see Table 1. For Sicilian samples, both COO and EVOO were put together.

According to the health claim for olive oil, authorized by European Union, a minimum of 250 mg kg^−1^ of selected phenols (hydroxytyrosol and its derivatives) is reported [27]. Figure 1A shows the content of these phenols in the studied olive oil samples. All the samples analyzed showed an average hydroxytyrosol, and its derivatives, content in accordance with the EU regulation [27]. As recommended by EFSA guidelines [6] a daily consumption between 10–20 g of EVOOs analyzed in this work ensures a daily intake of about 4–17 mg of oleuropein complex and hydroxytyrosol. It can also be seen that the nutraceutical value of COOs was lower than EVOOs, but more stable with smaller variations between samples, which may be related to technological factors such as the method of olive pressing or storage conditions. The only exceptions were the samples from Liguria, characterized by the lowest concentration of bioactive substances. Therefore, it is very important to control the quality of olive oil samples of both industrial and small-scale production.

During the research, in addition to phenolic alcohols and secoiridoids, phenolic acids and flavonoids were also determined; although they are not required for EU health claims, a proper assessment is needed since they contribute to increasing the health-promoting value of olive oil. Figure 1B shows the relative percentage of four chemical groups in EVOOs and COOs samples. It is relevant to note that the proportions of phenolic acids and flavonoids are similar in both oil groups. In the case of vitamin E, COOs, Sicilian and Ligurian oils were characterized by a higher relative content, while for secoiridoids it was the opposite. In both groups, the greatest fraction is represented by hydroxytyrosol and its derivatives (72–89%).

Tocochromanols or tocols belong to the group of tocopherols and tocotrienols described as vitamin E. These chemical compounds are fat-soluble antioxidants which inhibit lipid oxidation in various plant food products [28,29]. The major tocopherol found in olive oil is α-tocopherol, representing around 90% all isomers [30]. In this study, four chemical compounds, namely: α-, β-, γ-tocopherol and α-tocotrienol (their sum presented as vitamin E), were analyzed.

Table 2 summarize the tocopherol isomers and α-tocotrienol contents for EVOO and COO samples produced in Italy. The average vitamin E content in EVOOs and COOs was 169.0 ± 37.7 mg kg^−1^ and 151.7 ± 27.4 mg kg^−1^, respectively. The major quantified isomer was α-tocopherol, representing 90.8% and 88.0% of total vitamin E respectively, in the range from 70.2 to 232.2 mg kg^−1^ of oil, followed by lower amounts of β (1.6–11.5 mg kg^−1^) and γ isomers (1.3–17.7 mg kg^−1^) as well as α-tocotrienol (0.9–5.8 mg kg^−1^).

Comparable levels of tocopherols were mentioned by Saini et al. [28] and in USDA, ARS, FoodData Central database (https://fdc.nal.usda.gov/, accessed on 25 May 2020) [31]. The average content of α- and γ-tocopherol in olive oil was 143.5 and 8.3 mg kg^−1^, respectively. It can be observed that some olive oil samples from different regions were characterized by lower values of these parameters. In the case of EVOOs, Liguria and Garda had a lower value of both parameters, while in the case of COOs, it should be noted that in samples designated as oils extracted from Sicilian and Italy + EU olives low content of γ-tocopherol was determined. In this study, no significant differences in the content of α-, β-, γ-tocopherol and α-tocotrienol between EVOO and COO were found (*p* < 0.05) (Table S1, Supplementary material). Therefore, tocopherols, tocotrienols and total vitamin E cannot be considered as a discriminant marker of olive oil quality, which is in agreement with Fiorini et al. who stated that the tocopherol concentration did not differ significantly between high- and low-quality Italian extra virgin olive oil [20].

Phenolic acids are a group of hydrophilic phenols in olive oil. They contribute to the color and organoleptic properties but also to the antioxidant and health properties of food [3]. Many external factors can affect the content of this chemical class in extra virgin olive oils, such as harvesting time, olive fruit ripeness, climatic conditions and oil extraction technologies [32]. For this reason, it was also decided to study this chemical class in EVOOs and COOs. Gallic acid was chosen as the representative of this phenolic group. The average phenolic acid content was 1.3 ± 1.7 mg kg^−1^ and 6.5 ± 3.7 mg kg^−1^ for EVOOs and COOs respectively. Gallic acid concentration was higher in commercial samples than in oils purchased from family farms. It should be noted that among EVOOs more than 90% of the samples contained less than 4 mg kg^−1^ of phenolic acids, and in the case of COOs, it was less than 20%. However, as much as 30% of COOs had a result higher than 10 mg kg^−1^ of gallic acid. The results for EVOOs were consistent with earlier literature data for monovarietal extra virgin olive oil samples [33]. However, the differences in average concentrations of gallic acid between EVOOs and COOs were not statistically significant (Table S1, Supplementary material), therefore the concentration of phenolic acids cannot be a factor differentiating the sample due to the technology of their extraction.

Tyrosol (Tyr) and hydroxytyrosol (HTyr) are the main phenolic alcohols found in olive oil. It should be mentioned that hydroxytyrosol is the first phytochemical compound approved by EFSA as exhibiting antioxidant properties and the resulting health benefits [6]. The total concentration of phenolic alcohols was from 35.3 to 216.9 mg kg^−1^ for EVOOs and from 70.7 to 296.0 mg kg^−1^ for COOs. The results showed that the concentrations of the target compounds differed in the range 22.8–199.8 mg kg^−1^ (Tyr) and 5.4–71.3 mg kg^−1^ (HTyr) for the studied EVOO samples, as well as in the range 37.8–208.1 mg kg^−1^ (Tyr) and 20.7–124.3 mg kg^−1^ (HTyr) for the COO samples. It should also be noted that all samples contained more tyrosol than hydroxytyrosol, which is consistent with reports from other authors [23,34,35]. The phenolic alcohols results obtained in this study for Apulian and Tuscanian EVOOs were within ranges which were determined in samples of monovarietal olive oils from Apulia and Tuscany by Bellumori et al. [35]. Furthermore, the results obtained for both EVOOs and COOs were comparable to those for high-quality Italian extra virgin olive oils [23].

Based on Table 2, it can be seen that tyrosol concentrations fluctuated at similar levels in both types of oil, obtained from low-scale mills and commercially available. In the case of hydroxytyrosol and phenolic alcohols, COOs samples were characterized by a higher content of these substances. However, no significant differences were found between the average values of tyrosol, hydroxytyrosol and the content of phenolic alcohols (*p* < 0.05) between EVOOs and COOs (Table S1, Supplementary material), which eliminates them as indicators enabling distinguishing of these samples. In general, the phenolic alcohol content in fresh oil is low, but increases during storage due to hydrolysis of secoiridoids [36]. Hence, a higher content of this chemical class in COOs may result, because commercial samples may have been stored for longer or even stored in inappropriate conditions, whereby the hydrolysis has been accelerated.

Flavonoids, which are the dominant secondary metabolites of plants, were another hydrophilic class of phenols found in olive oil. The main flavonoids identified in the olive oil were flavones present mostly in free form, luteolin and apigenin. Flavonoids are also characterized by many bioactive properties; therefore, they can have beneficial effects in the case of cardiovascular diseases, neurodegenerative disorders or cancer [37].

Two flavonoids, luteolin and apigenin, were determined in the tested samples (Table 2). The total average concentration of this chemical class was 26.6 ± 13.5 mg kg^−1^ for EVOOs and 18.9 ± 7.6 mg kg^−1^ for COOs. The most common flavonoid was luteolin, with values ranging from 1.2 mg kg^−1^ to 53.0 mg kg^−1^. Apigenin concentration was lower than luteolin and determined values ranged from lower than LOQ to a maximum of 19.5 mg kg^−1^ for the EVOO sample from the Lazio region. According to Dugo et al. the content of luteolin in Italian high-quality extra virgin olive oil ranged from 4 to 149 mg kg^−1^, whereas apigenin from <LOD to 38.8 mg kg^−1^ [24]. The results obtained in this study were within this range, however, the average concentrations were lower than those determined by Dugo et al. [24]. These results confirm that flavonoids as well as phenol concentration are influenced by cultivars, the geographical variability (like climate and territory) and the different production techniques.

It can be observed that the oils from Sicily, both marked as high quality and commercially available, were characterized by low flavonoids content. In the remaining groups, apigenin and luteolin values remained at similar levels. No significant differences were found in the luteolin, apigenin or flavonoid content between the EVOO and COO samples (Table S1, Supplementary material). The flavonoid concentration cannot, therefore, be considered as an indicator discriminating the quality of olive oil.

These results confirm prior reports that flavonoids are a chemical class constituting a small part of the phenol fraction but characterized by relative concentration stability. This statement is consistent with de Torres, who proved that flavonoid concentrations depend mainly on the degree of olive ripeness [37]. Fiorini et al. proved that HEVOO and LEVOO (high-price and low-price extra virgin olive oils) were characterized by similar values of marked flavonoids [20]. Bakhouche et al., however, proved that the concentration of luteolin and apigenin did not differ significantly between Spanish olive oils from different geographical areas [38].

Secoiridoids are phenols which are usually glycosidically bound and formed from secondary terpene metabolism. They are the most prevailing chemical class in olive oil. Ligstroside and oleuropein aglycones and their decarboxymethylated forms, namely oleocanthal (DCL) and oleacin (DCO) are the most common secoiridoids in olive oils. Oleuropein aglycone and oleacin are hydroxytyrosol esters, while the other two are tyrosol derivatives [39,40]. Following the European Union recommendation, secoiridoids are important compounds in the health claim for olive oil [27].

All four above secoiridoids were determined in this study (Table 2). The average concentrations of this chemical class were 1530 ± 548 mg kg^−1^ and 546 ± 182 mg kg^−1^, for EVOOs and COOs, respectively. It should be emphasized that only one sample from the COO group contained more than 1000 mg kg^−1^ secoiridoids, while in the EVOO group as much as 65% of the samples showed this result. Except for one COO sample and two EVOO samples, the sum of aglycones was greater than the sum of DCO and DCL. Besides, hydroxytyrosol derivatives are more abundant than tyrosol derivatives in all cases, which is advantageous because hydroxytyrosol and its derivatives have higher antioxidant power than tyrosol [39]. Oleuropein aglycone was the most common secoiridoid in olive oil ranging from 39.5 to 1785.9 mg kg^−1^ for EVOOs and from 116.7 to 736.8 mg kg^−1^ for COOs. These results are in agreement with the outcomes of other authors using Italian and Spanish EVOOs [23,40].

Based on the analysis of variance and Tukey’s test, statistically significant differences were observed for mean concentrations of oleuropein aglycone and secoiridoids of EVOO and COO samples (*p* < 0.05) (Table S1, Supplementary material). In addition to the differences between samples produced in low-scale mills and industrial plants, it was also achievable to distinguish samples from different geographical areas. Based on the concentration of secoiridoids, it was possible to find differences between EVOO samples from 11 regions of Italy and create three groups characterized by different contents of bioactive compounds. The group with the lowest secoiridoids concentration included samples from Sicily and Liguria, without significant differences between them (*p* < 0.05), but significant differences among other samples, while the samples with the highest concentration of these compounds came from Abruzzo, Apulia, Campania, Sardinia and Tuscany. On this basis, the content of secoiridoids can be considered as a potential indicator of the quality or geographical origin of olive oil. This may be due to the fact that the profile of secoiridoids is influenced not only by climatic but also by technological factors [39,41].

### 3.1. Multivariate Statistical Analysis

The main purpose of the chemometric analysis was to reveal specific relationships between oil samples or between variables to classify extra virgin olive oils by phenolic content and indicate which variables were discriminatory chemical indicators for EVOOs and COOs. As chemometric methods, hierarchical cluster analysis (HCA), principal component analysis (PCA) and k-nearest neighbors (k-NN) algorithm were used.

#### 3.1.1. HCA (Hierarchical Cluster Analysis)

**Figure 2:**
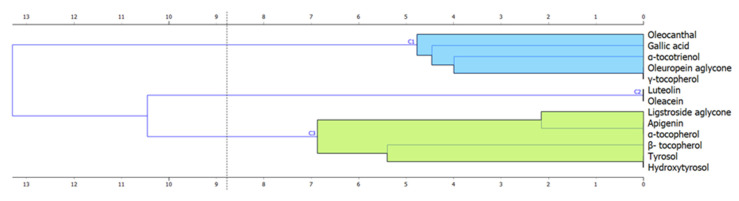
Hierarchical dendrogram for 13 chemical substances.

To understand the role of phenols as the quality indicators of extra virgin olive oils obtained from different places, HCA was used to group variables. Figure 2 presents a hierarchical dendrogram for 13 chemical substances. Based on the cluster analysis, it can be seen that phenols were grouped into three separate clusters based on the distance between data points in multidimensional space. In the case of reducing the number of variables, selecting variables from only one cluster should be avoided, as this may result in reduced model efficiency.

#### 3.1.2. PCA (Principal Component Analysis)

The phenolic profile was also used as data for processing by PCA for more comprehensive studies focusing on the identification of potential quality markers of extra virgin olive oils of various origins. The input set consisted of the concentrations of seven selected phenols, namely: gallic acid, β-tocopherol, oleuropein aglycone, ligstroside aglycone, oleacein, hydroxytyrosol and apigenin, which were selected based on ANOVA. Selected compounds belonged to all three clusters presented in the dendrogram (Figure 2).

**Figure 3:**
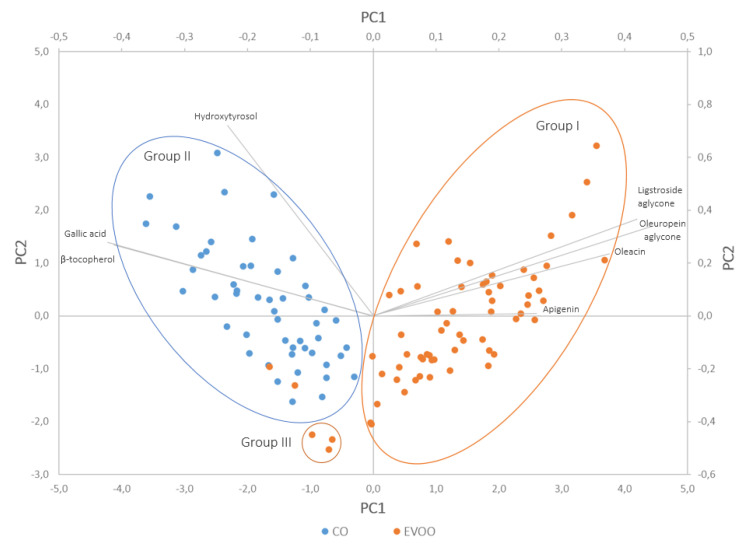
Bi-plot comparing scores along with loadings.

In Figure 3, bi-plot comparing scores along with loadings is depicted. The first two principal components explaining 63.1% of the total variance (45.2% and 17.9% for PC1 and PC2, respectively) divided the analyzed olive samples into three separate aggregations. The grouping of various extra virgin oils indicated that the qualitative and quantitative composition of phenolic compounds varies depending on both agronomic and technological factors. The division of samples into three subgroups was based on different contents of phenolic compounds. The first component (PC1) was positively correlated with chemical compounds from the class of secoiridoids and flavonoids. The other axis (PC2) was positively correlated with a representative of phenolic alcohols hydroxytyrosol. Interestingly, PC1 modelled the total polyphenol content with the richest oils on the right and the poorer ones on the left. This division allowed the separation of the first group containing only high-quality extra virgin olive oil samples. PC2 explained the difference between the second and third groups, namely, COO and EVOO, with the lowest content of phenol fraction (samples from Abruzzo, Lazio and Sicily). It can be observed that in the second group, apart from the COO samples, samples from Liguria were also grouped. The scatter plot with loadings described the behavior of variables, in other words, phenolic substances. It can be concluded that the levels of some compounds were positively correlated, for example, β-tocopherol and gallic acid, or oleuropein aglycone with ligstroside aglycone, with correlation coefficients higher than 0.5. Other substances were more negatively correlated, for example, apigenin with hydroxytyrosol or gallic acid.

The PCA-biplot allows correlation between the selected bioactive compounds and the groups of objects (olive oil samples). For example, secoiridoids (ligstroside aglycone, oleuropein aglycone, oleacein) and flavonoids (as apigenin) were positively correlated with high-quality extra virgin olive oil samples. It was therefore proved, that high-quality oils were characterized by a higher concentration of secoiridoids and flavonoids. In turn, phenolic alcohols (as hydroxytyrosol), phenolic acids, gallic acid and β-tocopherol were associated with commercial olive oils and lower quality extra virgin olive oils.

#### 3.1.3. k-NN (k-Nearest Neighbors)

To verify whether it is possible to assess the origin of extra virgin olive oil from small farms and supermarkets using concentrations of previously determined compounds as input variables for statistical analysis, k-NN was used to classify olive oils into two groups, namely EVOO and COO. EVOOs were the high-quality extra virgin olive oils samples from low-scale mills, while COOs were the lower-quality extra virgin olive oils which corresponded to commercially available extra virgin olive oils from industrial plants.

Input data were features previously selected for PCA. The data set was divided into two subsets, a training set and a test set. The accuracy of the classification model was measured using its recognition ability, in other words, the ability to classify samples from the training set, and the prediction ability, in other words, the ability to correctly classify samples from the test set. Through 10-fold cross-validation, it was found that the overall classification accuracy of the k-NN algorithm was 99%. The k-NN algorithm was then trained using 70% of the data selected at random to avoid bias and tested on the rest of the data. The use of k-NN resulted in an average of 94% correct classification with two false-negative results; this means that two samples of oils labelled as high-quality were classified as samples of lower quality. It should be noted that during multiple sampling, false-negative results were most common in samples from Liguria, Garda area or Sicily. During the PCA analysis, these samples were assigned to the second and third groups because they were characterized by a lower content of polyphenols compared to the samples from the first group (EVOO). It can, therefore, be concluded that by using the k-NN model, the quality of olive oil can be assessed, since samples classified as EVOO will have a higher nutraceutical potential compared to COO samples.

The model was also used to classify oil samples based on other features. According to Fiorini et al. [20], the classification was made applying the R parameter, in other words, the ratio of the concentration of tyrosol and hydroxytyrosol to the concentration of the above phenolic alcohols together with secoiridoid derivatives, the predictive efficiency was 85%. However, using HTyr and its derivatives, according to EFSA’s health statement [6,26], the classification was 88%. Referring to point 3.2.5., secoiridoids were also used as input, resulting in 91% correct classification.

Based on the results of multivariate statistical analysis, it can be assessed that for the extra virgin olive oil quality index the most relevant approach was to select the concentrations of seven selected phenols, namely: gallic acid, β-tocopherol, oleuropein aglycone, ligstroside aglycone, oleacein, hydroxytyrosol and apigenin.

## 4. Conclusions

This work aimed to evaluate the concentration of potentially bioactive compounds in both commercial and locally-produced Italian extra virgin olive oils. Lipophilic and hydrophilic phenolic compounds were analyzed by HPLC coupled with fluorescence, photodiode array and mass spectrometry detection. According to the quantitative data attained, for both types of EVOO samples, the phenolic content exceeded the nutritional claims, reported in the EFSA Journal [5,6]. The results achieved highlight how the European standards guarantee an EVOO product of high quality; on the other hand, smaller olive oil producers manage to distinguish themselves in the production of EVOOs with a higher secoiriodoid and hydrotyrosol content. Extraction technology from olives was found as a parameter which could influence the chemical composition of EVOOs, and in particular the bioactive molecule content. So differences in antioxidant molecule composition not only depend on cultivar, quality, territory, climate, storage conditions and olive maturity index but also from production techniques.

The present study could pave the way for a future more extensive “comprehensive” work with a larger array of EVOO samples produced beyond the European community.

## Figures and Tables

**Table 1:** High quality and commercial ^§^ extra-virgin olive oils analyzed.

Country	Denomination	Label	*Cultivar*, Year (n. Samples)
Apulia	PDO	Terra di Bari	*Coratina*, 2019 (1)
	MV		*Peranzana*, 2019 (2)
			*Olivastra*, 2019 (1)
			*Coratina*, 2019 (2)
			*Picholine*, 2019 (1)
	Blend		*Coratina-Peranzana*, 2019 (1)
Sicily	PDO	Monti Iblei	*Tonda Iblea-Moresca*, 2018 (1)
		Valle del Belice	*Nocellara del Belice*, 2018 (1) ^§^
		Val di Mazara	*Biancolilla-Cerasuola-Nocellara del Belice*, 2018 (2) ^§^
		Valli Trapanesi	*Biancolilla-Cerasuola-Nocellara del Belice*, 2018 (1) ^§^
	PGI		*Cerasuola*, 2018 (1) ^§^
			*Nocellara-Biancolilla-Cerasuola*, 2018 (3) ^§^
			*Nocellara Etnea*, 2018 (1) ^§^
			*Nocellara del Belice*, 2018 (1) ^§^
	MV		*Nocellara del Belice*, 2019 (1)
			*Nocellara Etnea*, 2018 (1) ^§^
Tuscany	PDO	Chianti classico	*Moraiolo*, 2019 (2)
			*Leccino-Moraiolo-Frantoio*, 2019 (3)
			*Moraiolo-Frantoio*, 2019 (2)
	PGI		*Frantoio*, 2019 (1)
			*Leccino-Moraiolo-Frantoio*, 2019 (5)
			*Leccino-Moraiolo-Frantoio- Pendolino*, 2019 (1)
	Bio		*Leccino-Moraiolo-Frantoio*, 2019 (5)
			*Moraiolo*, 2019 (2)
			*Frantoio*, 2019 (2)
	Blend		*Leccino-Moraiolo-Frantoio-Pendolino*, 2019 (2)
	MV		*Frantoio*, 2019 (1)
Liguria	PDO	Riviera Ligure	*Taggiasca*, 2019 (1)
	MV		*Taggiasca*, 2019 (1)
Campania	MV		*Leccio del Corno*, 2019 (2)
Abruzzo	PDO	Colline Teatine	*Gentile di Chieti-Intosso-Leccino*, 2018 (1)
			*Dritta*, 2019 (1)
			*Intosso*, 2019 (2)
	Blend		*Gentile di Chieti-Intosso-Leccino*, 2019 (2)
Garda area	PDO	Garda Trentino	*Casaliva-Leccino-Frantoio*, 2019 (1)
	MV		*Casaliva*, 2019 (2)
	Blend		*Casaliva-Frantoio-Leccino*, 2019 (1)
Calabria	Bio		*Carolea*, 2019 (1)
	MV		*Ottobratica*, 2019 (1)
	Blend		*Ottobratica-Sinopolese*, 2019 (1)
Lazio	Bio		*Canino*, 2019 (1)
			*Fratoio*, 2018 (1)
	MV		*Itrana*, 2018 (1)
			*Leccino*, 2019 (1)
			*Rosciola*, 2018 (1)
	Blend		*Caninese-Frantoio-Maurino-Leccino-Pendolino*, 2019 (1)
Sardinia	PDO	Sardegna	*Bosana-Semidana*, 2019 (1)
			*Bosana*, 2019 (1)
	Blend		*Bosana-Frantoio-Semidana-Coratina-Leccino*, 2019 (2)
	MV		*Bosana*, 2019 (2)
Umbria	Blend		*Leccino-Frantoio-Moraiolo*, 2019 (1)
			*Leccino-Frantoio-Moraiolo-S.Felice*,2019 (1)
Italy	Blend	EVOO	Not reported, 2018 (15) ^§^
EU	Olives from EU member states	EVOO milled in Italy	Not reported, 2018 (19) ^§^
Italy + EU	Olives from Italy + EU member states	EVOO milled in Italy	Not reported, 2018 (4) ^§^

^§^: commercial extra virgin olive oil samples; PDO: protected designation of origin; PGI: protected geographical identification; Bio: organic farming; MV: monovarietal.

**Table 2:** Content (mg kg^−1^) of bioactive molecules in olive oil samples analyzed divided into selected country/region.

		α-Tocopherol	α-Tocotrienol	α-Tocopherol	α-Tocopherol	Gallic Acid	Hydroxytyrosol	Tyrosol	Apigenin	Luteolin	Oleocanthal^a^	Oleacin^a^	Ligstroside Aglycone^b^	Oleuropein Aglycone ^a^
Apulia	range	166.9–220.6	0.9–3.1	3.1–8.7	2.8–16.0	<LOD–6.5	22.8–146.5	25.5–71.3	0.1–11.7	5–24.8	<LOD–11.8	180.3–566.3	101.6–513.1	343.4–1248.9
	average	195.4	2.4	4.1	10.7	1.7	70.4	41.3	4.5	16.2	7.6	352.1	298.9	903.6
Sicily	range	80.4–184.0	1.3–3.7	2.9–9.2	2.7–15.5	1.7–19.7	27.5–80.8	5.4–79.5	<LOD–8.5	1.4–34.0	1.8–30.5	34.0–396.2	5.7–78.4	139.5–418.4
	average	123.7	1.8	5.5	5.4	7.6	62.8	37.3	2.0	16.1	8.0	148.6	44.3	274.6
Tuscany	range	113.1–232.2	1.1–4.9	1.6–7.1	3.4–17.1	<LOD–9.3	25.4–199.8	12.5–59.9	<LOD–14.0	7.6–53.0	<LOD–38.7	35.1–493.4	47.4–329.4	268.7–1514.0
	average	152.9	2.4	2.7	11.4	1.4	78.3	30.8	4.4	25.9	5.9	229.2	172.4	872.6
Lazio	range	164.8–211.4	3.3–4.4	2.8–8.6	6.1–15.2	<LOD–4.3	34.9–80.4	8.2–52.0	<LOD–19.5	3.5–26.9	0.1–17.0	71.4–331.4	11.0–141.0	89.5–1749.5
	average	190.3	3.9	5.4	10.9	1.3	59.4	27.5	7.2	16.3	4.7	197.0	81.8	830.7
Abruzzo	range	99.5–198.1	1.7–3.4	2.3–4.5	2.3–13.2	<LOD–1.7	50.5–117.3	8.9–34.2	<LOD–7.1	9.3–34.4	1.9–34.8	123.0–590.9	20.4–370.8	138.7–1785.9
	average	129.6	2.7	3.1	5.6	0.8	95.0	19.9	1.7	16.8	14.3	225.6	114.9	896.0
Campania	range	153.5–198.8	2.6–2.7	3.0–3.8	9.2–17.7	0.4–3.1	111.3–168.8	41.7–45.4	1.1–2.0	16.2–19.9	1.3–31.2	59.7–498.7	131.8–254.3	877.8–915.7
	average	176.2	2.6	3.4	13.4	1.7	140.0	43.5	1.5	18.1	16.2	279.2	193.0	896.8
Garda	range	89.6–123.9	2.1–2.6	1.8–2.0	3.8–6.3	0.2–3.1	68.3–76.7	33.2–54.8	0.5–8.0	21.0–40.9	5.3–8.8	156.4–290.7	86.7–130.5	438.3–1015.1
	average	112.6	2.3	1.9	5.1	1.5	70.9	40.2	3.2	30.6	7.3	239.1	107.7	799.6
Sardinia	range	113.4–139.6	2.6–3.0	2.1–3.2	4.7–8.3	<LOD–0.7	39.1–69.5	19.2–42.4	<LOD–4.2	13.5–32.2	3.8–8.9	154.6–272.4	110.7–288.0	590.8–1105.4
	average	130.5	2.8	2.6	5.7	0.2	55.0	30.3	1.5	20.8	6.2	213.1	165.4	778.0
Calabria	range	144.0–201.0	3.5–3.9	3.4–6.7	3.7–16.4	<LOD–0.2	56.7–145.3	25.3–52.4	6.0–15.4	20.8–32.8	0.4–14.8	54.7–408.2	86.0–169.0	562.7–1004.1
	average	163.7	3.7	5.1	10.4	0.1	105.2	38.2	9.9	28.7	7.7	287.3	116.6	777.9
Umbria	range	130.6–156.1	2.4–2.4	1.8–2.4	11.0–11.8	<LOD–0.4	47.3–52.8	21.2–24.4	6.2–6.9	26.7–36.9	2.6–4.7	137.0–254.0	81.8–97.8	666.5–892.2
	average	143.4	2.4	2.1	11.4	0.2	50.1	22.8	6.6	31.8	3.7	145.5	89.8	779.3
Liguria	range	70.2–125.9	2.1–2.9	3.5–5.3	4.3–4.7	3.2–5.1	122.2–129.3	37.2–37.8	1.1–2.3	21.0–23.7	2,4–2.7	28.2–32.0	39.5–45.3	102.6–143.0
	average	98.1	2.5	4.4	4.5	4.1	125.8	37.5	1.7	22.3	2.6	30.1	42.4	122.8
Italy	range	84.5–151.4	1.6–4.0	3.9–10.2	3.9–13.9	2.3–13.3	47.7–145.2	38.6–124.2	<LOD–3.5	11.4–26.4	0.3–15.1	23.2–259.2	21.1–136.5	211.1–692.3
	average	128.0	2.8	6.5	9.1	6.5	84.1	59.7	1.2	18.1	5.3	137.2	68.3	434.7
EU	range	90.1–209.0	1.5–4.3	3.9–11.5	6.8–17.5	0.8–11.0	37.8–208.1	21.5–90.7	<LOD–6.1	8.5–27.0	0.3–5.4	22.4–177.0	21.1–142.9	218.7–736.8
	average	137.9	3.0	8.4	10.8	5.7	78.4	52.4	0.9	17.5	2.0	104.8	45.6	378.0
Italy + EU	range	152.7–165.9	1.6–5.8	6.1–10.4	3.1–12.9	2.3–14.5	53.1–93.2	20.7–82.7	<LOD–4.4	19.2–22.9	0.7–2.7	64.4–94.9	27.1–59.8	116.7–619.9
	average	164.3	3.2	8.6	10.7	5.1	71.0	55.7	2.1	21.3	1.9	70.2	40.8	422.8

Bioactive molecules were quantitatively determined based on calibration curves obtained with the correspondent standard compound: ^a^ oleuropein, ^b^ verbascoside.

## Supplementary Materials

The following are available online at https://www.mdpi.com/2304-8158/9/8/1120/s1, Table S1: Concentration of selected phenols in the extra virgin olive oils.
