# Supplementary material for: Concentration of Potentially Bioactive Compounds in Italian Extra Virgin Olive Oils from Various Sources by Using LC-MS and Multivariate Data Analysis
Source: Foods. 2020 Aug 13;9(8):1120. doi: 10.3390/foods9081120 (PMC7466375; doi:10.3390/foods9081120)
Supplement: Supplementary file 1 [file foods-09-01120-s001.pdf]

Table S1 Concentration of selected phenols in the extra virgin olive oils

No	Chemical compound/group	Concentration ± SD [mg/Kg]	
		EVOO	COO
1	α-tocopherol	153.5 ± 34.3	133.5 ± 25.0
2	α-tocotrienol	2.7 ± 0.9	2.6 ± 1.1
3	β-tocopherol	3.3 ± 1.7	7.1 ± 2.4
4	γ-tocopherol	9.5 ± 4.5	8.6 ± 3.8
5	Vitamin E	169.0 ± 37.7	151.7 ± 27.4
6	Gallic acid	1.3 ± 1.7	6.5 ± 3.6
7	Phenoic acids	1.3 ± 1.7	6.5 ± 3.6
8	Hydroxytyrosol	31.6 ± 13.4	50.2 ± 21.6
9	Tyrosol	77.5 ± 35.6	75.2 ± 27.0
10	Phenolic alcohols	109.1 ± 41.5	125.4 ± 41.4
11	Apigenin	4.1 ± 4.1	1.3 ± 2.0
12	Luteolin	22.4 ± 10.6	17.6 ± 6.0
13	Flavonoids	26.6 ± 13.5	18.9 ± 7.6
14	Oleocanthal[a]	7.4 ± 8.4	4.5 ± 5.2
15	Oleacin[a]	237.9 ± 129.7	123.0 ± 62.8
16	Ligstroside aglycone	158.8 ± 98.8	51.3 ± 27.7
17	Oleuropein aglycone	819.9 ± 404.4[a]	367.2 ± 141.2[a]
18	Secoiridoids	1224.1 ± 529.3[b]	546.0 ± 182.4[b]

Averages in rows marked with the same letters are significantly different by Tukey HSD test at $P<0.05$.
